# Resistance of *Anopheles gambiae* s.s. against commonly used insecticides and implication of cytochrome P450 monooxygenase in resistance to pyrethroids in Lambaréné (Gabon)

**DOI:** 10.1186/s12879-024-10021-y

**Published:** 2024-10-30

**Authors:** Stravensky Térence Boussougou-Sambe, Ynous Djida, Ange Gatien Doumba-Ndalembouly, Barclaye Ngossanga, Lynda Nouage Boussougou, Maminirina Fidélis Ambinintsoa, Rodrigue Bikangui, Daniel Nguiffo-Nguete, Francis N. Nkemngo, Romuald Agonhossou, Romaric Akoton, Jacques Dollon Mbama Ntabi, Abel Lissom, Francine Ntoumi, Charles S. Wondji, Peter G. Kremsner, Benjamin Mordmüller, Steffen Borrmann, Ayôla A. Adegnika

**Affiliations:** 1 Centre de Recherches Médicales de Lambaréné, Lambaréné, P.O. Box 242, Gabon; 2 Institut für Tropenmedizin, Eberhard-Karls-Universität, Wilhelmstrasse 27, Tübingen, P.O. Box 72076, Germany; 3 Centre for Research in Infectious Diseases (CRID), P.O. Box 1359, Yaoundé, Cameroon; 4 Fondation pour la Recherche Scientifique (FORS), P.O. Box 88, Cotonou, Benin; 5 Fondation Congolaise pour la Recherche Médicale, Brazzaville, Republic of the Congo; 6 Department of Zoology, Faculty of Science, University of Bamenda, Bamenda, Cameroon; 7 Department of Vector Biology, Liverpool School of Tropical Medicine, Pembroke Place, Liverpool, L3 5QA UK; 8 German Center for Infection Research (DZIF), Partner site Tübingen, Tübingen, Germany; 9 Department of Medical Microbiology, Radboudumc, Nijmegen, The Netherlands

**Keywords:** *A. gambiae s.s*., Pyrethroids, Resistance intensity, Cytochrome p450, Gabon

## Abstract

**Background:**

Insecticides are a crucial component of vector control. However, resistance constitute a threat on their efficacy and the gains obtained over the years through malaria vector control. In Gabon, little data on phenotypic insecticide resistance in *Anopheles* vectors are published, compromising the rational implementation of resistance management strategies. We assessed the susceptibility to pyrethroids, carbamates and organophosphates of *Anopheles gambiae sensu lato* (*s.l.*) and discuss the mechanisms involved in the pyrethroid resistance-phenotype.

**Methods:**

*A. gambiae s.l.* larvae were collected from breeding sites in Lambaréné. Emerging adults were used in WHO tube assays at an insecticide concentration that defines resistance (diagnostic concentration). Subsequently, deltamethrin and permethrin were used at 5x and 10x diagnostic concentrations and after preexposure with the cytochrome p450 (and glutathione S-transferase) inhibitor piperonyl butoxide (PBO). A subset of mosquitoes was typed by molecular methods and screened using Taqman assays for mutations conferring target site resistance at the Voltage-gated sodium channel 1014 (*Vgsc-1014*) locus and the acetylcholinesterase (*Ace-1*) gene.

**Results:**

All mosquitoes were *A. gambiae sensu stricto* (*s.s.*) and resistant to permethrin, deltamethrin and alphacypermethrin (mortality less than 98%). However, mosquitoes were susceptible to malathion but resistant to bendiocarb. The level of resistance was high for permethrin and at least moderate for deltamethrin. Pre-exposure to PBO significantly increased the mortality of resistant mosquitoes (*P <* 0.0001). They became fully susceptible to deltamethrin and permethrin-induced mortality increased 4-fold. The G119S *Ace-1* resistance allele, which confers resistance to both organophosphates and carbamates, was not present. All sampled mosquitoes were either homozygous for the *Vgsc-L1014F* or heterozygous for *Vgsc-L1014F/L1014S*, a marker for resistance to pyrethroids and organochlorides.

**Conclusion:**

These findings demonstrate a role of cytochrome P450 monooxygenases in the pyrethroid-resistance of *A. gambiae s.s*. from Lambaréné. Combining PBO with pyrethroids, as done in second generation bednets, may be used to revert resistance. In addition, malathion could also be used in combination with pyrethroids-based methods for resistance management.

**Supplementary Information:**

The online version contains supplementary material available at 10.1186/s12879-024-10021-y.

## Introduction

 Insecticide resistance is a looming threat on the success of malaria vector control measures. Although, a large share of the reduction in malaria cases has been attributed to vector control measures [1], a stagnation in malaria cases has been observed since 2016. From 2020, the number of cases even increased [2] due on one hand, to the disruptions in the delivery of medication and diagnostics during the COVID-19 pandemic [2] and on the other hand, to the reduced impact of vector control measures at further decreasing the incidence and mortality of malaria.

Malaria vector control in Gabon, a country located in Central Africa, is based on the free provision of Long Lasting Insecticidal Nets to pregnant women and children. Although previous reports have shown the spread of pyrethroid resistance in most sub-Saharan African countries [3–7], in Gabon the data are still sparse with few reports showing resistance to pyrethroid [8, 9] with low intensity resistance to permethrin, deltamethrin and lambdacyhalothrin in agricultural areas of Mouila [8]. However, the investigation of resistance mechanisms to insecticides in Gabon has been limited to genotyping *A. gambiae* populations for markers of target site resistance, which is a resistance mechanism where a modification of the site of action of an insecticide is changed such that it no longer binds effectively, resulting in the insect being unaffected or less affected by the insecticide [10]. Most reports in Gabon have shown a near fixation of the *Kdr* resistance alleles that confer resistance to pyrethroids and organochlorides [11–15]. In contrast, a glycine to serine (G119S) amino acid substitution (*ace-1*^*R*^) in the mosquito’s acetylcholinesterase, conferring resistance to both organophosphates and carbamates was reported only at a minor fraction of mosquitoes in Libreville [12]. Subsequent studies found no evidence of the allele in other parts of the country [12–15] with only one study reporting full susceptibility of *Anopheles* populations in Mouila to organophosphates and carbamates published, so far.

Metabolic resistance, the most common resistance mechanism in insects [10], is characterized by changes in a mosquito’s enzyme system which result in a more rapid detoxification or catabolism of the insecticide, reducing the insecticide’s concentration at its site of action [16]. In the case of malaria vectors, three enzyme families are known to be important insecticide metabolizers: cytochrome P450 monooxygenases, glutathione S-transferases and esterases. Resistant strains have been shown to possess higher levels or more efficient forms of these enzymes than susceptible counterparts [17] in Cameroon [18] and Tanzania [19]. Cytochrome P450 oxidases are most commonly involved in resistance, metabolizing a wide range of insecticides [17]. Cytochrome P450-mediated resistance mechanism have been reported in many *Anopheles* species across sub Saharan Africa (sSA) including *A. gambiae s.s*., *A. coluzzii* and *A. funestus* [6, 20–25]. However, no reports have assessed metabolic resistance in *Anopheles* populations in Gabon. Gabon is planning to carry its first massive distributions of Long Lasting Insecticidal Nets (LLINs) in the coming years. In the context of reports of *Anopheles* resistance to insecticides at the molecular [11–15] and phenotypic [8, 9] levels, it is pivotal to collect data on the susceptibility of malaria vectors to pyrethroids and assess the effect of metabolism modifiers that can act synergistically with insecticides to revert resistance to pyrethroids as a potential resistance management strategy. The current study therefore aimed to evaluate the susceptibility profile of mosquitoes to pyrethroids, organophosphates and carbamates, to assess the level of resistance and the effect of piperonyl butoxide (PBO), a synergistic compound used to revert pyrethroid resistance.

## Methodology

### Study area and mosquito collections

**Fig. 1:**
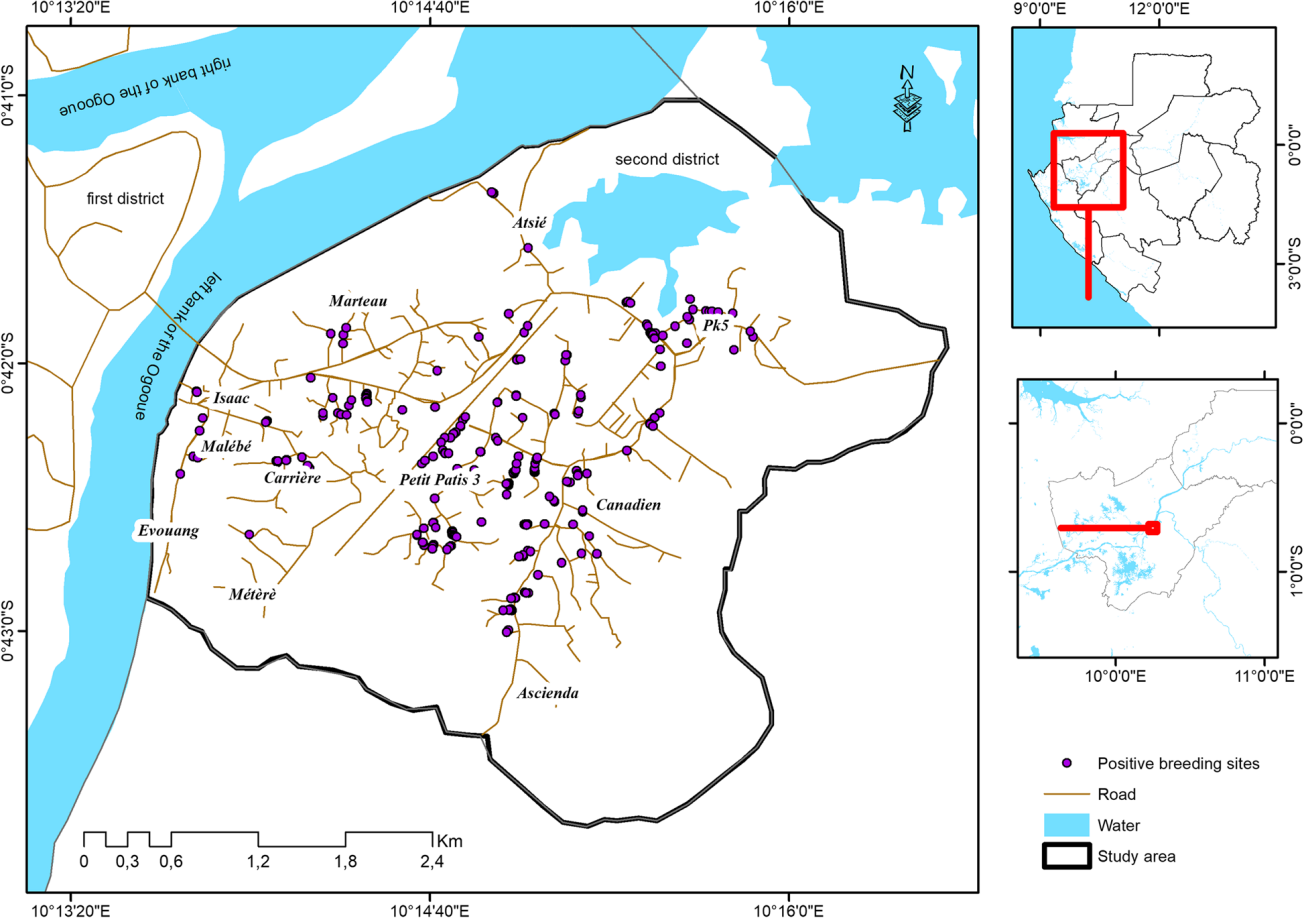
Map of the study area

The study was conducted in the second district of Lambaréné, the capital of the Moyen Ogooué province from December 2021 to May 2022 (Fig. 1). Lambaréné is a semi-urban settlement that is divided in three parts by the Ogooué River and is surrounded by forests. The city is also neighboured by two extensive agricultural schemes of palm and rubber trees. The second district is the most populated district of the city. Previous studies in the city and its surrounding villages have shown that *A. gambiae s.s.* is the main malaria vector [15, 26, 27] with heterogenous malaria transmission intensity with perennial transmission in some areas while in others, no transmission was recorded during the dry season. In addition, previous reports have shown resistance of *A. gambiae s.s.* from this area to deltamethrin, permethrin and DDT [9].

Larvae were collected from breeding sites and reared up to the adult stage at 29 ± 1 °C under 12 h dark:12 h light cycle at the Medical Entomology Laboratory of the Centre de Recherches Médicales de Lambaréné (CERMEL). Emerging mosquitoes were provided with 10% sugar solution and kept at 26 °C ± 1 °C and 80 ± 10% relative humidity until the day of the test.

### WHO susceptibility assays

The tests were carried out using impregnated papers with the following concentrations: deltamethrin (0.05%), permethrin (0.75%), alphacypermethrin (0.05%), bendiocarb 0.1% and malathion 5% following the WHO test tube protocol for adult mosquitoes [16]. These concentrations represent the threshold concentrations that discriminate the proportions of susceptible and resistant phenotypes in a sample of a mosquito population (“diagnostic concentration”) [16]. We tested the impregnated papers with the Kisumu strain reared at the CERMEL before the tests with field collected mosquitoes to confirm the quality of the papers. All the tests were performed in controlled conditions at 26 °C ± 1 °C and 80% ± 10% relative humidity.

Briefly, three-to-five-day old unfed *Anopheles gambiae s.l.* mosquitoes were exposed in WHO susceptibility kits to impregnated papers with insecticides while controls mosquitoes were exposed to untreated filter papers. The number of mosquitoes knocked down was recorded at different time intervals (5, 10, 15, 20, 30, 40, 50 and 60 min). After 1 h of exposure, mosquitoes were transferred to observation tubes and were maintained on a 10% sugar solution for 24 h. Mortality was recorded after a 24-hour recovery period. The mosquitoes were stored on silica gel for molecular assays. Mortality in the field collected unexposed controls was less than 4%.

### Assessment of resistance intensity

Based on the results from the susceptibility tests with standard diagnostic concentration, resistance intensity to pyrethroids was assessed using 5X and 10X the diagnostic concentrations for permethrin and 5X the diagnostic concentration for deltamethrin. The test procedure was the same as described above with knockdown recorded over a 1-hour period and mortality assessed after 24 h following WHO protocol [16] .

### Piperonyl butoxide synergist tests

Resistance bioassays tests were performed with the PBO synergist which inhibits the activity of cytochrome P450 monooxygenases in order to assess the involvement of metabolic resistance. Briefly, three-to-five-day old, starved *Anopheles gambiae s.l.* mosquitoes were pre-exposed to 4% PBO for one hour and then exposed to 0.05% deltamethrin and 0.75% permethrin for another hour. The number of mosquitoes knocked down was recorded over a 1-hour period as described above. The mosquitoes were then transferred to observation tubes and maintained on a 10% sugar solution for 24 h. Mortality in the PBO-only exposure group was less than 4%.

### Molecular identification

DNA was extracted from control for the screening of knockdown mutations which are already fixed in local anopheles populations [15] and from susceptible and resistant mosquitoes to bendiocarb using the Livak protocol [28]. The members of the *A. gambiae* complex were identified using the *SINE200* protocol [29]. Taqman assays were used to screen mosquitoes using previously published protocols for *Kdr* mutations (*Vgsc-L1014F* and -*L1014S*) [30] and *Ace-1* mutation [31]. For both Taqman assays, 1.0 µl of DNA template was amplified using 5 µl SensiFAST™ Probe No-ROX Kit (Meridian Bioscience Inc.), 0.8 µl forward (10 µM) and 0.8 µl reverse (10 µM) primers, 0.2 µl of each probe (10 µM) and 2 µL of nuclease free water to a final volume of 10 µl.

### Statistical analysis

We used the WIN DL (version 2.0) software [32] to determine the different times required to knockdown 50% (KDT_50_) and 95% (KDT_95_) of the samples for each insecticide tested with a log-time probit model.

The results of the tests were interpreted based on the WHO guidelines [16] with mortality above 98% indicating susceptibility, while 90–97% mortality indicating potential resistance while mortality less than 90% interpreted as resistance. Low resistance intensity was defined as 98–100% mortality at the 5× concentration (but < 90% at 1×). Mortality < 98% at the 5 × concentration and 98–100% at the 10× concentration indicated moderate resistance intensity. Mortality < 98% at the 10× concentration indicated high resistance intensity [16].

The differences in mortality between insecticides tested were compared using the Fisher’s exact and the chi-square tests. Graphical presentation of data was done using the GraphPad Prism Version 8.4.0 software (GraphPad Software Inc.). In addition, we also tested the distribution of genotypes for conformity to Hardy Weinberg Equilibrium (HWE).

## Results

### Species composition, genotyping of *Kdr* and *Ace-1* mutations

Out of the 140 *A. gambiae s.l* that were identified molecularly, 97.9% (137/140) were successfully typed as *A. gambiae s.s.* using the *Sine200* protocol.

All the 94 mosquitoes screened for the presence of the knockdown resistance genes were either homozygous (98.4%) for *Vgsc-L1014F* or heterozygous (1.6%) for *Vgsc-L1014F* and *Vgsc-L1014S.* The distribution of genotypes was in HWE equilibrium (χ^2^ = 0.02, df = 1, *P =* 0.8764). By contrast, all the mosquitoes screened for the *Ace1* mutation were carrying the G119 *Ace-1* susceptible allele of the gene.

### Insecticide susceptibility

**Fig. 2:**
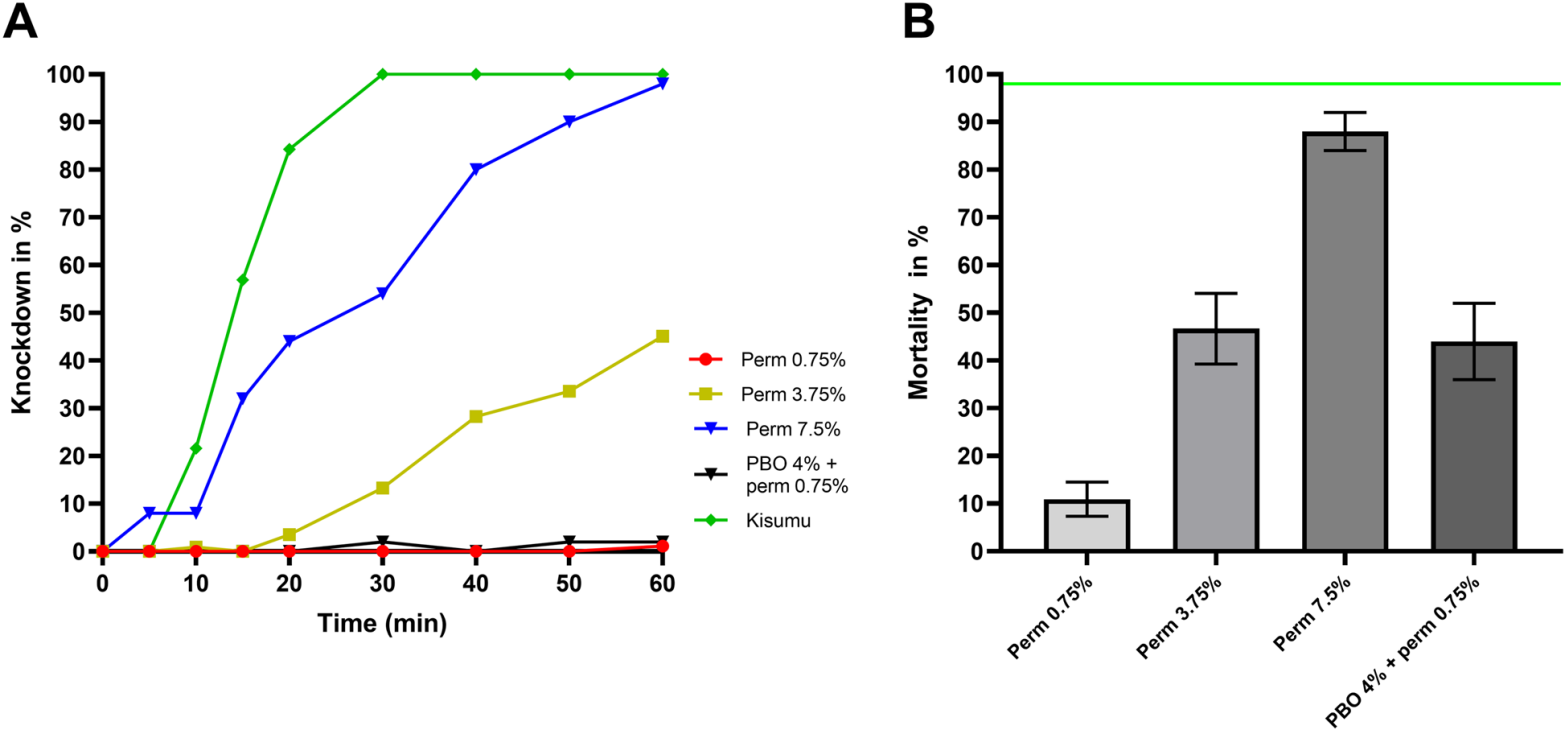
Susceptibility profile of *A. gambiae s.s.* to permethrin only and after preexposure to PBO. **A** Proportion of mosquitoes knocked down after exposure to various concentrations. **B** Mean mortality after exposure to various concentrations. The horizontal green line represents the 98% WHO threshold for susceptibility. Error bars represent standard error of mean mortality after the 24h recovery period following insecticide exposure

*A. gambiae s.s.* mosquitoes were resistant to 0.75% permethrin with a mortality of 11.4% (10/88) after the 24-hour observation period (Fig. 2B). There was a total loss of knockdown effect of permethrin on *A. gambiae s.s.* individuals tested; with only one (1) mosquito out of eighty-eight (88) knocked down after one hour exposure to the diagnostic concentration of permethrin (Fig. 2A). Therefore, no KDT_50_ nor KDT_95_ could be calculated after exposing the mosquitoes to the diagnostic concentration of permethrin.

**Fig. 3:**
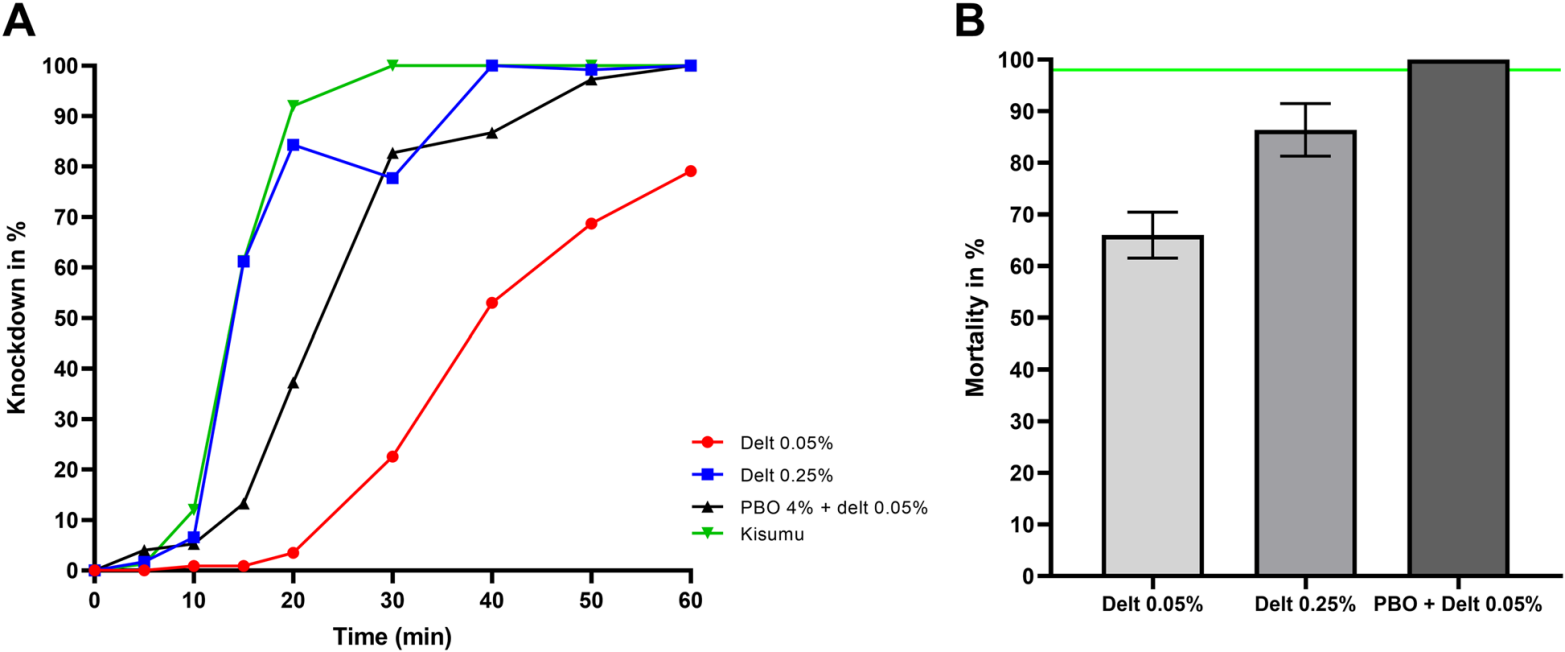
Susceptibility profile of *A. gambiae s.s.* to deltamethrin only and after preexposure to PBO. **A** Proportion of mosquitoes knocked down after exposure to various concentrations. **B** Mean mortality after exposure to various concentrations. The horizontal green line represents the 98% WHO threshold for susceptibility. Error bars represent standard error of mean mortality after the 24h recovery period following insecticide exposure. Proportion of mosquitoes knocked down after exposure to various concentrations of deltamethrin

Mosquitoes were more susceptible to deltamethrin in comparison to permethrin (χ^2^ = 61.14, df = 1, *P <* 0.0001). However, they were still resistant to deltamethrin with a 66.1% (76/115) mortality after 24 h (Fig. 3B). Although, a knockdown effect was observed with deltamethrin (Fig. 3A), there was still a 2.9-fold increase in the KDT_50_ of field populations (40.9 min) compared to the susceptible Kisumu strain (13.9 min) (Table S1) while the KDT_95_ was above 60 min.

**Fig. 4:**
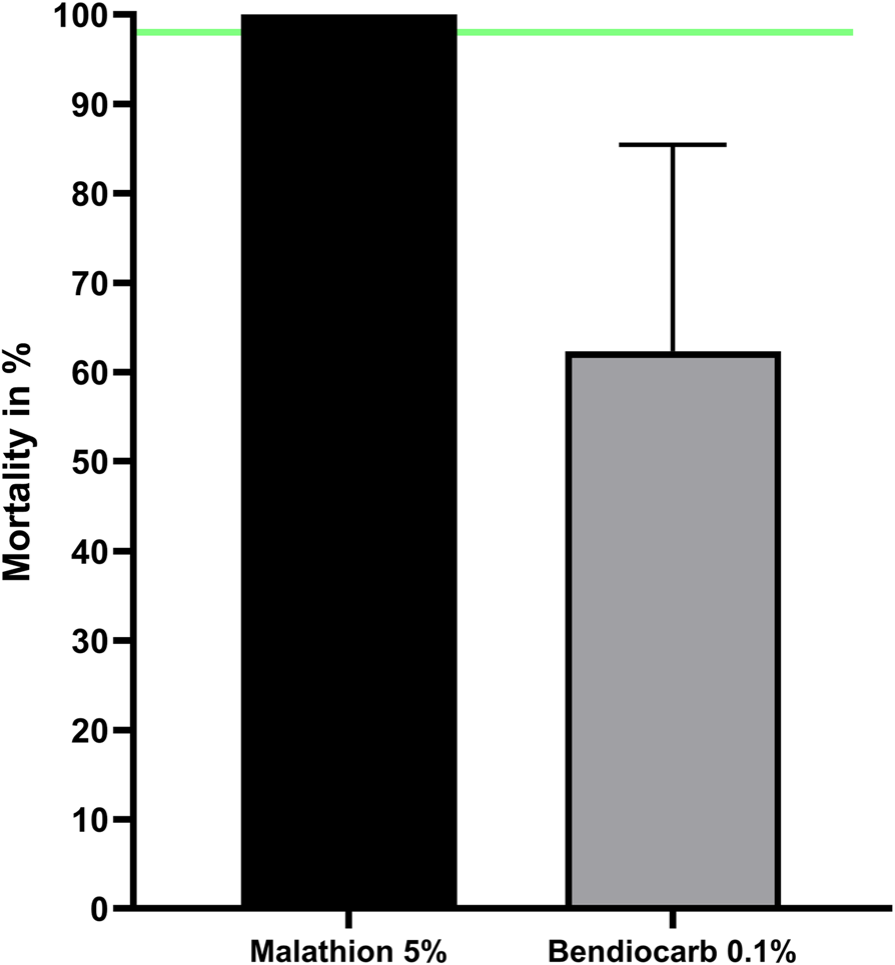
Mean mortality of *A. gambiae *s.s. after exposure to diagnostic concentrations of malathion and bendiocarb. The horizontal green line represents the 98% WHO threshold for susceptibility. Error bars represent standard error of mean mortality after the 24h recovery period following insecticide exposure

*A. gambiae s.s.* were also resistant to 0.05% alphacypermethrin with a mortality of 45.8% (11/24) and both KDT_50_ and KDT_95_ above 1 h (Table 1). However, mosquitoes were fully susceptible to malathion with 100% (75/75) mortality whilst they were resistant to bendiocarb 0.1% with a mortality of 62.7% (47/75) after the 24-hour recovery period (Fig. 4).

**Table 1:** Knockdown times and mortality of A. gambiae s.s. from Lambaréné after insecticide susceptibility tests

Insecticides tested	*N*	KDT_50_(min) [CI_95_]	Rtkd_50_ [CI_95_]	KDT_95_ (min) [CI_95_]	Rtkd_95_ [CI_95_]	Mortality (%)
Per 0.75%	88	No kd	NA	No Kd	NA	11.4
PBO + Per 0.75%	50	No Kd	NA	No Kd	NA	44
Per 3.75%	113	63.6 [57.4–73.2]	4.7	187.1 [143.4–278.6]	7.7	47.8
Per 7.5%	50	24.1 [20.7–27.2]	1.8	64.3 [54.5–81.8]	2.6	88
Del 0.05%	115	40.9 [38.9–43.1]	2.9	81.4 [73.5–93.0]	3.8	66.1
Del 0.25%	121	15.3 [9.6–19.7]	1.1	33.9 [25.3–76.4]	1.6	84.3
PBO + Del 0.05	75	23.2 [21.4–24.9]	1.7	44.2 [40.2–49.9]	2.1	100
Alpha 0.05	24	65.5 [55.2–97.2]	4.8	149.7 [99.7–463.1]	6.2	45.8

### Resistance intensity

Resistance intensity was only assessed for permethrin and deltamethrin. The resistance intensity to permethrin was high with mortalities below 98% at both 5 × (47.7%, 53/111) and 10 × (88%) the diagnostic concentrations (Fig. 2B). Although we observed an increased number of mosquitoes knocked down after one hour (51 out of 113), the resulting KDT_50_ and KDT_95_ at 5X the diagnostic concentration was above 60 min. Meanwhile, the KDT_50_ at 10X the diagnostic concentration was 24.1 min, a 1.8-fold increase in comparison to the Kisumu strain. The KDT_95_ for the mosquitoes from Lambaréné was above one hour (Fig. 2A).

For deltamethrin tests were conducted with 5X the diagnostic concentration only because the number of available larvae was low. Mosquitoes displayed a moderate resistance intensity to deltamethrin with a mortality of 84.3% at 5X the diagnostic concentration (Fig. 3A). Both KDT_50_ (15.3 min) and KDT_95_ (33.9 min) with 0.25% deltamethrin were below 1 h with a 1.6-fold increase for the latter compared to the susceptible Kisumu strain (Table 1).

### Synergist assays

Synergist assays were performed with both permethrin and deltamethrin. The mortality in the PBO only group was 4% (2/50). Preexposure to PBO led to a total recovery of the susceptibility of *A. gambiae s.s.* to deltamethrin (66.1% mortality with 0.05% deltamethrin vs. 100% mortality with PBO + 0.05% deltamethrin; Fisher’s exact test *P <* 0.0001) (Fig. 3B). The recovery of full susceptibility following preexposure to deltamethrin was accompanied by a decrease in KDT_50_ (40.9 min for deltamethrin 0.05% vs. 23.2 min with PBO + deltamethrin 0.05%) and KDT_95_ (> 1 h for deltamethrin 0.05% vs. 44.2 min with PBO + deltamethrin 0.05%) (Table 1). PBO and permethrin led to a 4-fold increase in mortality compared to permethrin alone (11% with 0.75% permethrin vs. 44% with PBO + permethrin, Fisher’s exact test, *P <* 0.0001) (Fig. 2B). Interestingly, the knockdown capacity of the insecticide was not improved: only one (1) mosquito knocked down after exposure to this insecticide.

## Discussion

Insecticide resistance has spread all over sSA and there is a need to monitor the pattern of its spread to ensure the implementation of suitable insecticide resistance management strategies.

From the current study, we found that *A. gambiae s.s* from Lambaréné were more resistant to permethrin than deltamethrin, with a loss of knockdown effect of permethrin and fold increases in knock down times for deltamethrin as similarly reported from previous studies in Gabon [8, 9]. However, mosquitoes were less resistant to deltamethrin when comparing the current results with previous ones from Lambaréné [9] which could be due to a reduction in the selection pressure resulting from the absence of large-scale deployment of vector control measures suspected to select for resistance in *Anopheles* mosquitoes [33, 34]. In addition, as Lambaréné is surrounded by two extensive agricultural schemes where pesticides are not used, suggest that the use of pesticides by gardeners and the use of insecticides in households could be the main drivers of pyrethroid resistance. Mosquitoes were also resistant to alphacypermethrin, as also reported in other sSA countries [35, 36], and we report the first evidence of resistance to this insecticide in Gabon. The full susceptibility observed with malathion suggests that it could be used with pyrethroids in combination strategy. This strategy is based on the assumption that mosquitoes that will not be killed by one, will be killed by the other insecticide [37, 38]. However, the resistance observed to bendiocarb is worrying as it limits the pool of insecticides available for resistance management in Gabon.

Preexposure to PBO lead to fold increases in mortality of *A. gambiae s.s.*, as shown elsewhere [23–25], to both permethrin and deltamethrin with a full restoring of susceptibility to the latter. However, preexposure to PBO did not lead to the restoration of the knockdown effect of permethrin whilst a decrease in knockdown times was observed with deltamethrin. The knockdown effect in addition to the excito-repellency of pyrethroids are key features that make them suitable for bed nets impregnation, as they allow the nets to remain efficient even when they are torn [39]. The fact that preexposure to PBO increased the mortality of *A. gambiae s.s.* populations suggests that metabolic resistance, with an overexpression of P450 monooxygenases enzymes, is primarily responsible for the insecticide resistance phenotype [19, 40]. However, this does not exclude the involvement of other metabolic enzymes such as esterases and glutathione S-transferases. These results suggest that PBO LLINs may be a better option for mass distribution in Lambaréné and presumably in Gabon as those nets have been shown to provide superior protection in areas with pyrethroid resistant mosquitoes [41, 42].

There was a high resistance intensity to permethrin in *A. gambiae s.s.* and at least a moderate resistance intensity to deltamethrin contrary to results obtained in Mouila where a low resistance intensity to pyrethroids was recorded [8]. The difference with the aforementioned study points to the need for susceptibility testing in different regions of the country to have an overview of the resistance profile of malaria vectors in Gabon. However, the 5-year time gap between the two studies carried out respectively in 2017 and 2022 does not exclude an escalation of the resistance intensity in the meantime. According to the WHO criteria [16] our results point to the potential risk of operational failures of vector control measures based on the use of these two insecticides in Lambaréné, especially for permethrin. Current recommendations suggest that remedial action must be implemented in such cases with the use of synergists as a potential mitigation measure.

All the mosquitoes were carrying the knockdown resistance *Vgsc- 1014 F* and − *1014 S* alleles which is already fixed in the local *A. gambiae* population as previously reported in Gabon [11–15]. However, we found a lower proportion of the *Vgsc-1014 S* alleles compared to previous reports which could be due to a fitness cost associated with carrying this allele and to the fact that it may offer a lower protection against pyrethroids compared to the *Vgsc-1014 F* [43]. Despite the resistance to bendiocarb, no mosquitoes were found carrying the G119S *Ace-1* resistance allele in accordance with previous publications in Gabon where it was reported either absent [14, 15] or present at a low level [12] which points to the involvement of metabolic resistance to bendiocarb. Similar results were reported in Chad where despite high resistance to bendiocarb no G119S *Ace-1* mutation was found [44] contrary to results from Cameroon where resistance to this insecticide was strongly correlated to the presence of G119S Ace-1 mutation [45]. The main limitations of this study are the small sample size used for the tests with alphacypermethrin as well as the fact that we did not specifically determine the enzymes involved in metabolic resistance using transcriptional analyses.

## Conclusion

*A. gambiae s.s.* populations from Lambaréné were resistant to permethrin, deltamethrin and alphacypermethrin. Here, we showed that mosquitoes were highly resistant to permethrin and at least moderately resistant to deltamethrin. This high level of resistance intensity especially for permethrin constitutes a serious threat for the mass distribution of LLINs. From our results, the combination of both PBO and deltamethrin should be considered for LLINs distribution in Lambaréné and the surrounding areas by the National Malaria Control Programme. The full susceptibility to malathion qualifies it as a welcome addition to the toolbox for the management of insecticide resistance in Lambaréné. It will be interesting to test its susceptibility pattern systematically in Gabon.

## Supplementary Information

Supplementary Material 1.

## Data Availability

Raw data are archived and available on request from the corresponding author.

## Abbreviations

*Ace*: acetylcholinesterase
CERMEL: Centre de Recherches Médicales de Lambaréné
kdr: Knockdown resistance gene
KdT: Knockdown time
LLINs: Long Lasting Insecticidal Nets
PBO: piperonyl butoxide
sSA: sub–Saharan Africa
*Vgsc*: Voltage-gated Sodium Channel
