# Supplementary material for: Resistance of Anopheles gambiae s.s. against commonly used insecticides and implication of cytochrome P450 monooxygenase in resistance to pyrethroids in Lambaréné (Gabon)
Source: BMC Infect Dis. 2024 Oct 30;24:1221. doi: 10.1186/s12879-024-10021-y (PMC11523776; doi:10.1186/s12879-024-10021-y)
Supplement: Supplementary file 1 — Supplementary Material 1. [file 12879_2024_10021_MOESM1_ESM.docx]

| Insecticides tested | N | KDT_50_(min) [CI_95_] | KDT_95_ (min) [CI_95_] | Status |
| --- | --- | --- | --- | --- |
| Per 0.75% | 51 | 13.7 [12.6 – 14.8] | 24.3 [21.7 – 28.6] | Susceptible |
| Del 0.05% | 75 | 13.9 [13.1 – 14.7] | 21.3 [19.7 - 23.9] | Susceptible |
| Alpha 0.05% | 51 | 13.7 [12.6 - 14.8] | 24.3 [21.7 - 28.6] | Susceptible |

Table 1: Knockdown times of *A. gambiae* s.s*.* (Kisumu)
